# Deleted in Liver Cancer 2 (DLC2) Was Dispensable for Development and Its Deficiency Did Not Aggravate Hepatocarcinogenesis

**DOI:** 10.1371/journal.pone.0006566

**Published:** 2009-08-10

**Authors:** Tai On Yau, Thomas Ho Yin Leung, Sandra Lam, Oi Fung Cheung, Edmund Kwok Kwan Tung, Pek Lan Khong, Amy Lam, Sookja Chung, Irene Oi Lin Ng

**Affiliations:** 1 Liver Cancer and Hepatitis Research Laboratory and SH Ho Foundation Research Laboratories, Department of Pathology, The University of Hong Kong, Hong Kong, China; 2 Department of Diagnostic Radiology, The University of Hong Kong, Hong Kong, China; 3 Department of Anatomy, The University of Hong Kong, Hong Kong, China; University of Florida, United States of America

## Abstract

DLC2 (deleted in liver cancer 2), a Rho GTPase-activating protein, was previously shown to be underexpressed in human hepatocellular carcinoma and has tumor suppressor functions in cell culture models. We generated DLC2-deficient mice to investigate the tumor suppressor role of DLC2 in hepatocarcinogenesis and the function of DLC2 in vivo. In this study, we found that, unlike homologous DLC1, which is essential for embryonic development, DLC2 was dispensable for embryonic development and DLC2-deficient mice could survive to adulthood. We also did not observe a higher incidence of liver tumor formation or diethylnitrosamine (DEN)-induced hepatocarcinogenesis in DLC2-deficient mice. However, we observed that DLC2-deficient mice were smaller and had less adipose tissue than the wild type mice. These phenotypes were not due to reduction of cell size or defect in adipogenesis, as observed in the 190B RhoGAP-deficient mouse model. Together, these results suggest that deficiency in DLC2 alone does not enhance hepatocarcinogenesis.

## Introduction

Hepatocellular carcinoma (HCC) is a primary malignancy and is one of the most common cancers in Asia and Africa. Infection with hepatitis B or C virus infection and exposure to aflatoxin B1 have been well documented as the major causes. However, the underlying molecular mechanisms leading to the development and progression of HCC remain unclear.

Inactivation of tumor suppressor genes is a hallmark of cancer cells. In a quest for tumor suppressors involved in hepatocarcinogenesis, we identified a novel gene *DLC2* (deleted in liver cancer 2) on chromosomal region 13q12.3, which is frequently lost in HCCs [1]. Similar to its homolog DLC1, DLC2 is underexpressed in human HCCs and has tumor suppressor functions in cultured cells [1], [2].

DLC1 and DLC2 are Rho GTPase-activating proteins (GAPs). They have three functional domains, a catalytic RhoGAP domain [1], [3], a sterile alpha motif (SAM) protein interaction domain [4], [5] and a lipid-binding StAR-related lipid-transfer (START) domain [6], [7]. The Rho family GTPases have 18 members, including the three well-studied representatives, Rac1, RhoA, and Cdc42. These small GTPases regulate various cellular signaling pathways [8], [9]. It has been suggested that the Rho proteins play important roles in oncogenic transformation mediated by Ras and other oncoproteins by regulating actin cytoskeleton organization, cell proliferation and cell survival [10], [11]. Also, they stimulate tumor cell invasion and metastasis [9]. Rho GTPase-activating proteins (GAPs) inactivate them by converting the active GTP-bound state to the inactive GDP-bound one through activation of the intrinsic GTPase activity of Rho proteins. GAPs have therefore been suggested to be tumor suppressors which counteract the oncogenic potential of Rho proteins.

There have been several lines of in vitro evidence to support the tumor suppressor role of these two Rho GAP proteins, DLC1 and DLC2 [1], [2], [3], [5], [12], [13], [14], [15], [16]. Animal models which could be exploited to investigate the tumor suppressor functions of these two proteins are still limited. Durkin et al. [17] has generated a DLC1-deficient mouse model to study the biological functions of DLC1. However, the DLC1 deficiency leads to embryonic lethality by midgestation. Sordella et al [18] showed that embryos lacking p190B RhoGAP were about 30% smaller in size and provided evidence to suggest that this reduction in embryo size was due to reduction in cell size. They also demonstrated that murine embryonic fibroblasts derived from these embryos were defective in adipogenesis when compared with the wild type counterpart [19].

In this study, we generated DLC2-deficient mice to investigate the biological functions of DLC2 and its role in hepatocarcinogenesis in vivo. Unlike the DLC1 knockout model, DLC2 was dispensable for embryonic development and DLC2-deficient mice could survive to adulthood. DLC2-deficient mice did not show higher incidence of hepatocarcinogenesis or diethylnitrosamine (DEN)-induced hepatocarcinogenesis. However, DLC2-deficient mice were significantly smaller and had less adipose tissue than the wild type mice. Analysis of adipogenesis of DLC2-deficient and wild type murine embryonic fibroblasts did not show significant difference between them. Also, we did not observe any significant difference in the cell size between G1 phase DLC2-deficient and wild type immortalized fibroblasts with flow cytometry.

## Materials and Methods

### Generation of DLC2-deficient mice

**Figure 1:**
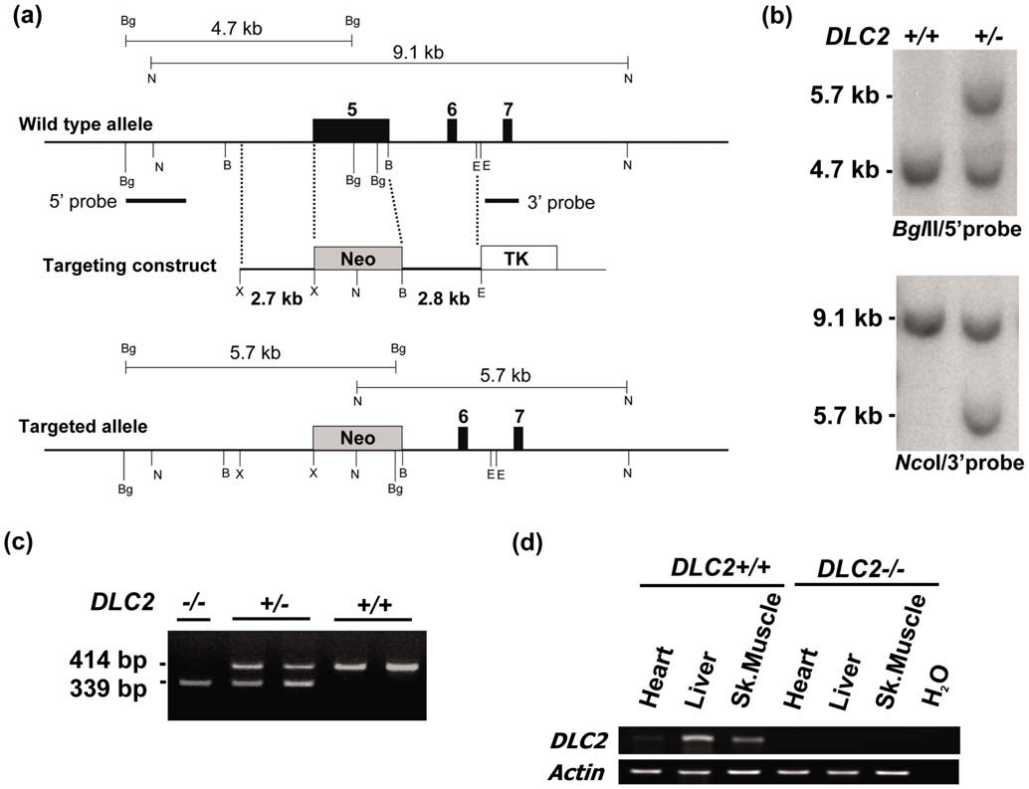
Gene targeting to generate DLC2-deficient mice. (a) Schematic diagram to show gene targeting strategy. Black boxes, exons of the mouse DLC2 gene (exon numbers are written at the top); gray boxes, DNA sequence of neomycin resistance gene of the targeting construct; white box, DNA sequence of thymidine kinase gene of the targeting construct. Restriction enzyme digestion sites: B, BamHI; Bg, BglII; E, EcoRI; N, NcoI; only diagnostic restriction sites are shown for simplicity. (b) Southern blot analysis. BglII digestion was probed with 5′ probe as indicated; sizes of diagnostic bands: wild type allele, 4.7 kb; targeted allele, 5.7 kb. NcoI digestion was probed with 3′ probe as indicated; sizes of diagnostic bands: wild type allele, 9.1 kb; targeted allele, 5.7 kb. (c) PCR genotyping; sizes of bands: wild type allele, 414 bp; targeted allele, 339 bp. (d) RT-PCR to detect DLC2 expression in heart, liver and skeletal muscle.

A PAC clone containing the mouse *Dlc-2* genomic region was obtained from the Sanger Centre (Cambridge, United Kingdom). To construct the mouse *Dlc-2* gene targeting vector, a 2.8 kb *Eco*RI/*Bam*HI fragment, which is 3′ to exon 5, was cloned into the *Eco*RI/*Bam*HI site of pPNT (Figure 1a). A pair of primers (forward, 5′-ttactcgagttgctgatgcacaggtcttc; reverse, 5′-ttactcgagttctcgtgttaggaatgggg) was used to amplify a genomic region, 2.7 kb in size and 5′ to exon 5 (Figure 1a). The fragment was then cloned into pPNT [20]. The targeting vector was linearized with *Not*I and was electroporated into the AB2.2 embryonic stem (ES) cell line (129s7/SvEBrd-Hprt b-m2), and the cells were cultured in ES cell culture medium supplemented with G418 and fialuridine. Resistant clones were analyzed by Southern blotting (see below) to identify *DLC2*-targeted clones.

All research involving animal work was approved by the Committee on the Use of Live Animals in Teaching and Research (CULATA) of the University of Hong Kong. Cells from these clones were injected into C57BL/6N blastocysts. Chimeras were mated with C57BL/6N mice, and offspring containing *DLC2*-targeted allele were identified. Mice with mixed genetic backgrounds were back-crossed to C57BL/6N for at least five generations.

### Southern blot analysis, PCR genotyping and RT-PCR

For Southern blot analysis, two probes, 5′ and 3′ external, were used (Figure 1a). *Bgl*II digestion was probed with the 5′ probe and the sizes of diagnostic bands were 4.7 kb for the wild type allele and 5.7 kb for the targeted allele. *Nc*oI digestion was probed with the 3′ probe, and the sizes of diagnostic bands were 9.1 kb for the wild type allele and 5.7 kb for the targeted allele.

Two pairs of primers were used for PCR genotyping. For detection of the wild type allele, forward (5′-AATGGAAGCACCATGTAGCC) and reverse primer 5′- AATGGAAGCACCATGTAGCC) were used, with expected size of the PCR product 414 bp. For detection of the targeted allele, forward (5′-CTGGGAAGACAGGGAACAAA) and reverse primers (5′-GGGGGAACTTCCTGACTAGG) were used, with expected size of the PCR product being, 339 bp.

For semi-quantitative RT-PCR analysis, total RNA was prepared from mouse tissues using the TRIzol reagent (Invitrogen). First-strand cDNA was synthesized from 1 µg of total RNA using random hexamers with GeneAmp RNA PCR Kit (Applied Biosystems, Foster City, CA). Two µl of cDNA was then used for detecting the expression levels of *DLC1, DLC2* and *DLC3* using the following primers: For mouse *DLC1* gene, forward primer (5′- CCCATTCAGTCAGTCCACCTTGA) and reversed primer (5′- GAGTCTCCCTCGTCGGAAAAC) were used; For mouse *DLC2* gene, forward primer (5′-GATGAGAAACACAGCCAGCA) and reverse primer (5′-GTTGGGTATCTGGACGCACT) were used; For *DLC3*, forward primer (5′-TCCACAGGCAGCCATGCCAG) and reversed primer (5′-TCTTGCTCAAGATCCTGGGCC) were used. PCR condition was as follows: denaturation at 95°C for 15 min, followed by 25 cycles (*DLC1* and *DLC2*) or 27 cycles (*DLC3*) of denaturation for 30 sec, annealing at 55°C for 30 sec with extension at 72°C for 30 sec and a final extension at 72°C for 10 min. PCR products were resolved in 1.2% agarose gel.

### Metabolic cage experiment

Mice were individually housed in metabolic cages in a 12∶12-hour dark-light cycle and were fed *ad libitum*. Water and food consumption, and urine and faeces output were measured for 2 days.

### Diethylnitrosamine (DEN) treatment of mice

The hepatocarcinogenesis protocol was as follows. The 15-day-old male *DLC2* homozygous knockout mice and wild type mice were injected intraperitoneally with 25 mg/kg DEN in sterile PBS. At 35 weeks of age, mice were sacrificed and their livers were harvested and examined. The livers were then fixed in 4% formaldehyde in PBS, embedded in paraffin and cut into 4-µm sections for histological examination.

### Preparation of murine embryonic fibroblasts (MEFs) and establishment of cell lines

Pregnant mice were sacrificed at 13.5 days past coitus (dpc). The embryos were dissected from the uterus, and the extra-embryonic membranes and viscera were removed. The embryos were cut into small pieces, and soaked for 30 min in 4 ml of 0.25% trypsin-EDTA at room temperature. Trypsinization was inactivated with α-modified Eagle medium (αMEM) supplemented with 10% heat-inactivated FBS, 50 U of penicillin per ml, and 50 µg of streptomycin per ml. The cells were suspended by pipetting and passed through a strainer. Standard procedures were used to establish immortalized fibroblast cell lines [21]. Briefly, cells were passaged every 3 days in DMEM supplemented with 10% calf serum and antibiotics at a density of 10^6^ cells per 10 cm dish. Media were changed on the subsequent day. Stable immortalized cell lines were established after 30 to 50 passages.

### Measurement of cell sizes

The relative sizes of *DLC2^+/+^* and *DLC2^−/−^* cells were determined with flow cytometry. Briefly, cells were BrdU-labeled using FITC BrdU Flow Kit (BD Pharmingen™, NJ, USA). G1 population of cells was gated and the forward light scatter (FSC) of 5000 G1-cells was determined. FSC was used to determine the relative cell sizes.

### Induction of adipocyte differentiation

For induction of adipocyte differentiation, MEF cells used were within two passages. Induction of adipocyte differentiation was performed as previously described [19]. Cells were cultured on 6-well plates and propagated to confluence. Two days later, the medium was replaced with standard differentiation induction medium (α-MEM containing 0.5 mM isobutylmethylxanthine, 1 µM dexamethasone, 5 µg of insulin per ml, 10% FBS, 50 U of penicillin per ml, and 50 µg of streptomycin per ml, and the medium was renewed every other day. The cells were induced for 8 days before analysis.

The cells were fixed in 10% formalin, rinsed twice with phosphate-buffered saline (PBS), and stained with Oil-Red O staining solution (0.5% Oil-Red O in isopropyl alcohol solution-distilled water [60∶40]) for 30 min at 37°C and then washed with distilled water three times. Adipogenesis in Oil-Red-O-stained MEF was quantified by measuring the absorbance of light at 510 nM after extraction with isopropyl alcohol.

### Rhotekin binding assay

Cells were lysed in 500 µl of lysis buffer containing 50 mM Tris (pH 7.4), 10 mM MgCl2, 500 mM NaCl, 1% Triton X-100, 0.1% SDS, 0.5% sodium deoxycholate, 10 µg/ml of aprotinin, 10 µg/ml of leupeptin, and 1 mM PMSF. The lysates were cleared by centrifugation at 12,000 × g for 10 min at 4°C, and 500 µg of each lysate was incubated with 45 µg of GST-RBD (GST fusion protein containing RhoA-binding domain of Rhotekin) bound to glutathione-Sepharose beads (Amersham Pharmacia) for 1 h. After binding, the samples were washed with lysis buffer for three times. Bound proteins were fractionated on 12% SDS/PAGE and immunoblotted with polyclonal antibody for RhoA (Santa Cruz Biotechnology). Total cell lysate was also analyzed with anti-RhoA antibody as a loading control. The level of active RhoA was determined after normalization with the total RhoA present in the cell lysates.

### Immunofluorescence staining

Cells were grown on cover slips and were rinsed in PBS, fixed with 4% paraformaldehyde in PBS for 15 minutes at room temperature, permeabilized with 0.5% Triton X-100 in PBS for 5 minutes and blocked with 5% bovine serum albumin in PBS. After washing with PBS, cells were then incubated with paxillin antibody (Sigma), followed by FITC-conjugated secondary antibody at room temperature. F-actins were stained with tetramethylrhodamine B isothiocyanate-labeled phalloidin (Sigma) for 20 min at room temperature, and the nuclei were stained by DAPI. Cells were mounted with Vectashield antifade solution (Vector Laboratories).

## Results

### Generation of DLC2-deficient mice

To study the function of the DLC2 *in vivo* and the role of DLC2 in hepatocarcinogenesis, we generated DLC2-deficent mice. Disruption the mouse DLC2 gene was achieved by homologous recombination in embryonic stem (ES) cells. In the recombination, exon 5, the largest exon of *DLC2* gene, was replaced by the neomycin resistance gene of the targeting vector (Figure 1a). Two targeted ES clones were used to establish the DLC2-deficient mice (Figure 1b and 1c). *DLC2^−/−^* mice did not express the *DLC2* mRNA as detected by RT-PCR on selected tissues, heart, liver and skeletal muscle (Figure 1d).

### DLC2-deficient mice were anatomically normal but smaller and had less adipose fat

**Figure 2:**
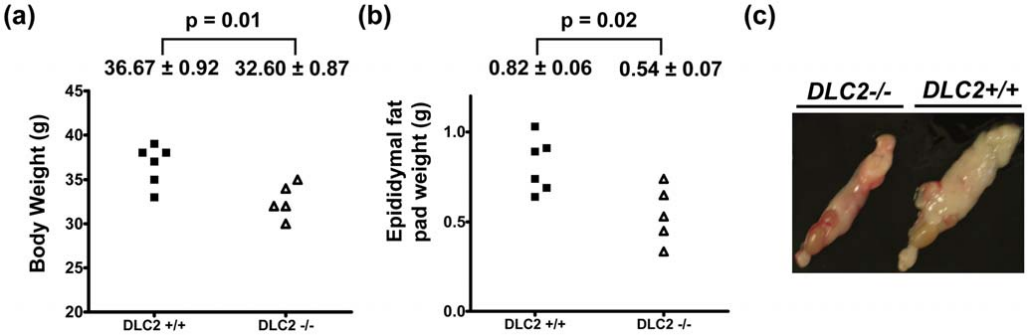
DLC2-dificient mice were smaller and had less adipose fat. (a) Body weight. (b) Weight of epididymal fat pads. In (a) and (b), mean values±standard errors and p-values are shown. P-values were calculated by unpaired Student's t test. (c) Representative samples of epididymal fat pads.

**Figure 3:**
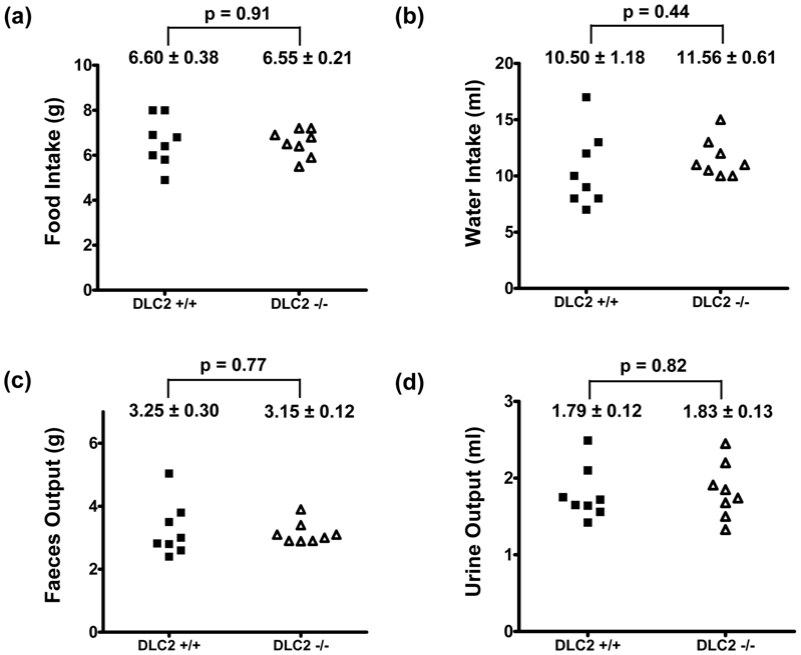
Food and water intake in DLC2+/+ and DLC2−/− mice. (a) Food intake, (b) water intake, (c) feces output and (d) urine output in 24 hours. Mean values±standard errors and p-values are shown. P-values were calculated by unpaired Student's t test.

Unlike the *DLC1^−/−^* mice, which died in embryonic stage, *DLC2^−/−^* mice could survive to adulthood. This suggests that DLC2 is dispensable for embryonic development. Anatomical analysis showed that *DLC2^−/−^* mice were normal (at 30 week-old), however *DLC2^−/−^* mice were smaller in size and lighter in weight (p = 0.01) (Figure 2a) and had less adipose tissue than wild type mice (p = 0.02) (Figure 2b & 2c). We wanted to exclude the possibility that *DLC2^−/−^* mice might eat less food than *DLC2^+/+^* mice and this might contribute to their smaller body size. We therefore performed metabolic cage experiment to investigate whether there were any differences in food and water consumption. However, we could not detect any significant differences in food and water intake between *DLC2^−/−^* mice and *DLC2^+/+^* mice (Figure 3).

### Depletion of DLC2 did not affect cell size and adipogenesis in MEFs

The p190B RhoGAP-deficient mouse embryos were smaller than the wild type embryos and fibroblasts derived from the p190B RhoGAP-deficient embryos were smaller than those derived from wild type embryos [18]. Also, fibroblasts derived from the p190B RhoGAP-deficient embryos were defective in adipogenesis [19]. We therefore examined whether fibroblasts derived from the *DLC2^−/−^* mouse embryos were smaller in size and defective in adipogenesis.

**Figure 4:**
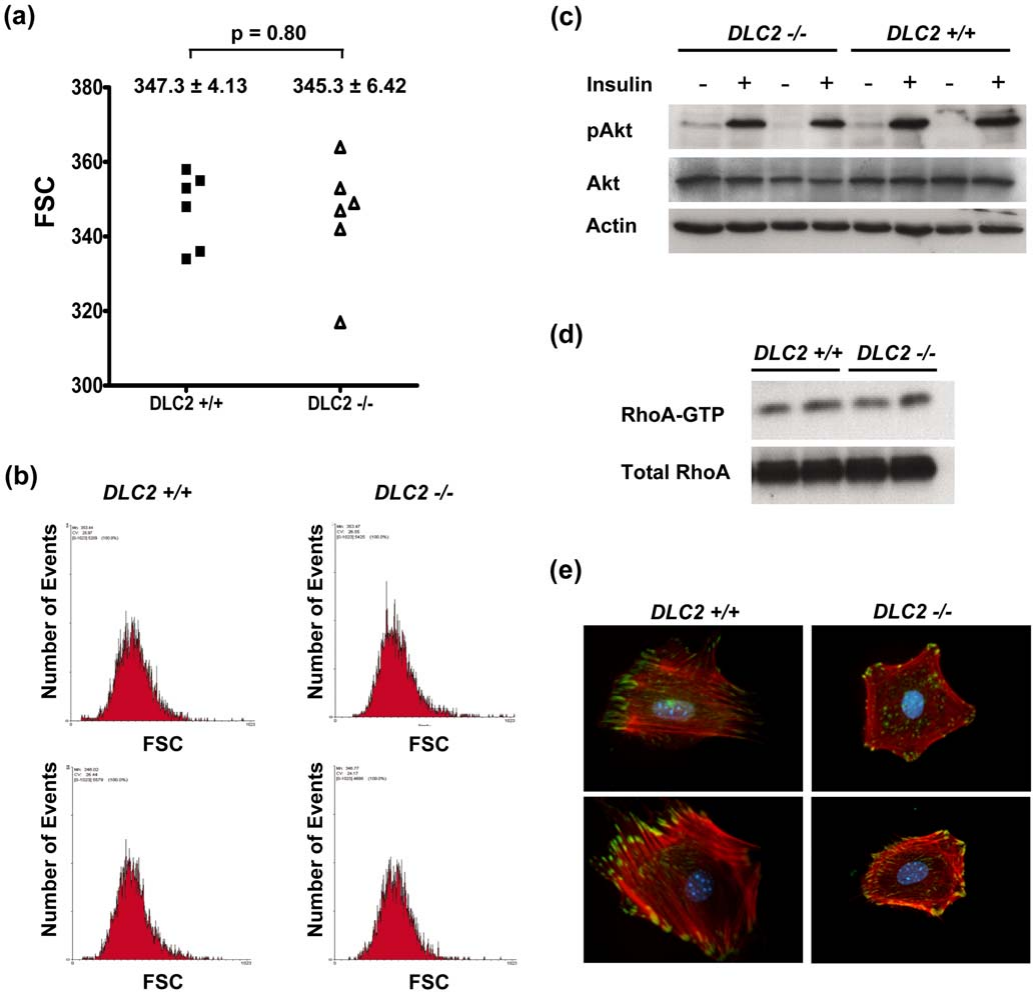
Depletion of DLC2 does not affect cell sizes. (a) Comparison of sizes of DLC2+/+ (n = 6) and DLC2−/− (n = 6) cells by flow cytometry. Forward light scatter (FSC) of G1-phase-cells as determined by flow cytometry was used to measure the relative cell sizes. Mean values±standard errors and p-values are shown. P-values were calculated by unpaired Student's t test. (b) Representative samples of flow cytometry results. (c) Western blot analysis of insulin-treated DLC2+/+ and DLC2−/− cells. (d) RhoA activity of DLC2+/+ and DLC2−/− cells as detected by Rhotekin binding assay. (e) Actin cytoskeleton and focal adhesions in DLC2+/+ and DLC2−/− MEF cells were detected by immunofluorescence staining of actin stress fibers (red) and Paxillin (green), respectively.

As demonstrated with flow cytometry, the size of the cells derived from the *DLC2^−/−^* mouse embryos was not significantly different from that of the *DLC2^+/+^* mouse embryos (Figure 4a and 4b). Since Sordella et al. [18] showed the AKT pathway was downstream target of RhoA, and this eventually led to the decreased cell size of fibroblasts derived from the p190B RhoGAP-deficient embryos, we examined the AKT pathway in cells derived from the *DLC2^−/−^* and *DLC2^+/+^* mouse embryos. We did not detect any difference between them in this pathway (Figure 4c). We previously showed that DLC2 was a RhoGAP; overexpression of DLC2 down-regulated Rho activity in hepatoma cell lines and resulted in inhibition of actin stress fiber formation [2]. However, up-regulation of Rho activity in the *DLC2^−/−^* cells was not observed (Figure 4d) and there was some slight but not significant difference in the formation of actin stress fibers or focal adhesions between *DLC2^−/−^* and *DLC2^+/+^* cells (Figure 4e). These suggest that there might be other functionally similar proteins which could compensate the function of DLC2.

**Figure 5:**
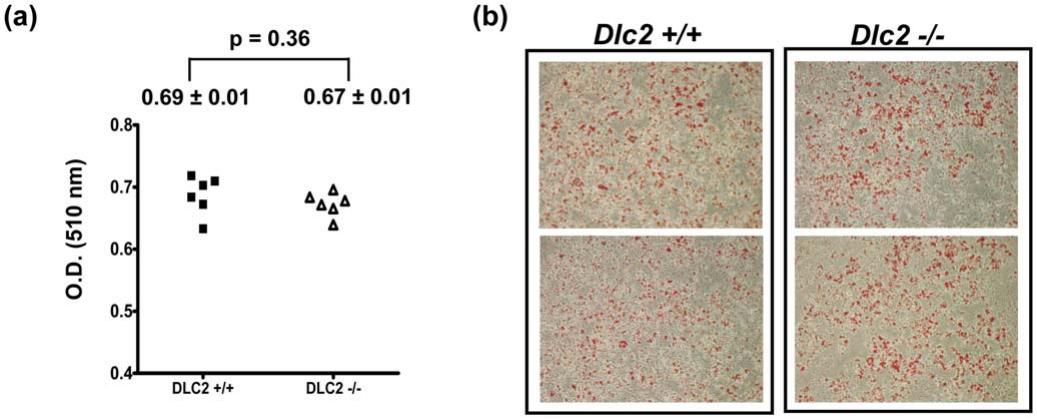
Depletion of DLC2 does not affect adipogenesis in MEFs. (a) Induction of adipogenesis was performed on DLC2−/− (n = 6) and DLC2+/+ (n = 6) MEFs. After induction, MEFs were stained with Oil-Red O, and the stain was then extracted and absorbance at 510 nm was measured. Mean values±standard errors and p-values are shown. P-values were calculated by unpaired Student's t test. (b) Representative samples of Oil-Red O-stained MEFs.

We then examined adipogenesis in fibroblasts derived from the *DLC2^−/−^* and *DLC2^+/+^* mouse embryos. When *DLC2^−/−^* and *DLC2^+/+^* cells were induced by standard adipocyte differentiation induction medium, both *DLC2^−/^* ^−^ and *DLC2^+/+^* cells were competent to differentiate into adipocytes and did not significantly differ in lipid production quantitatively (Figure 5).

### Depletion of DLC2 did not predispose to the formation of liver tumors nor aggravate DEN-induced hepatocarcinogenesis

**Figure 6:**
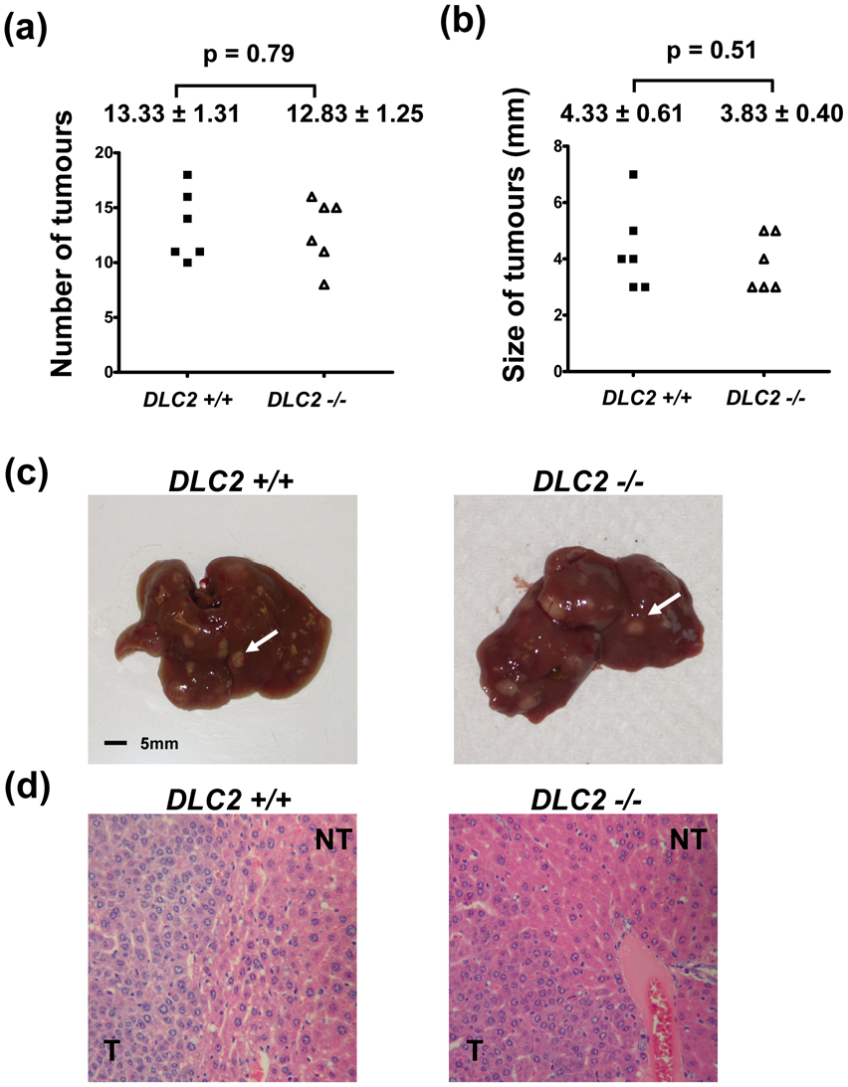
Depletion of DLC2 did not aggravate DEN-induced hepatocarcinogenesis. (a) Number of tumors formed in livers after DEN treatment. (b) Size of tumor as represented by maximum diameter (in millimeters). In (a) and (b), mean values±standard errors and p-values are shown. P-values were calculated by unpaired Student's t test. (c) Representative liver samples treated with DEN. White arrows indicate tumors. (d) Representative H&E stained sections of liver samples treated with DEN.

We did not observe any tumor formation in the livers of the adult *DLC2^−/−^* mice up to an age of 18 months. To address the role of DLC2 in chemical-induced hepatocarcinogenesis, we treated the 15-day old male *DLC2^−/−^* and *DLC2^+/+^* mice with DEN. When the mice reached 35 weeks of age, they were dissected and their livers were examined for tumor formation. We observed that liver tumors developed in all of the mice treated with DEN. In addition, the numbers of tumors formed and the maximal tumor diameters were similar in both the *DLC2^−/−^* and *DLC2^+/+^* mice (p = 0.79 and 0.51, respectively) (Figure 6). These findings suggest that DLC2 deficiency does not aggravate chemical hepatocarcinogenesis.

### *DLC1* and *DLC3* mRNA expression in DLC2-depleted tissues

Semi-quantitative PCR was performed on selected tissues including livers, skeletal muscles and hearts of the *DLC2^−/−^* and *DLC2^+/+^* mice at 3 months of age to observe any gene dosage compensation by other DLC members in our *DLC2^−/−^* mice. Our data showed no significant increase of either DLC1 or DLC3 in those tissues of the *DLC2^−/−^* mice (Supplementary Figure S1). The experiments were performed independently thrice.

## Discussion

It was reported that DLC1-deficient embryos had neural tube defects and died at early to midgestation [17]. As suggested by Durkin et al. [17], this might also be due to the primary malfunction of other organs such as the developing heart. Our group recently showed that DLC1 negatively regulated the Rho/ROCK [22]. Also, a relatively high level of ROCK expression was previously observed in the developing hearts of embryos, 7.0–9.0 dpc [23] and 9.5–11.5 dpc [24]. This suggests that DLC1 deficiency may disturb the normal function of ROCK proteins in the embryonic heart and lead to embryonic lethality. Nevertheless, the observed phenotypes suggest that DLC1 has a critical role during early murine development.

In this study, we showed that DLC2 deficiency did not cause any observable developmental defects, and DLC2-deficient mice could survive to adulthood. This suggests that the functions of DLC2 in embryonic development may be compensated by its homolog, DLC1, but not vice versa. It is common that paralogous genes compensate the functions of the members of the group. For example, the members of mouse Hox paralogous group 3, Hoxa3, Hoxb3 and Hoxd3, interact synergistically to regulate embryonic development in a dose-dependant manner [25], [26]. Although DLC2 deficiency did not lead to embryonic lethality, DLC2-deficient mice were smaller and had less adipose tissue. Since it was demonstrated that the fibroblasts derived from 190B RhoGAP-deficient embryos were smaller [18] and were less effective in adipogenesis [19], we examined the DLC2-deficient mice along these lines. However, we observed no significant differences in cell size and adipogenesis between fibroblasts derived from *DLC2^−/−^* and *DLC2^+/+^* mice. Also, the metabolic cage experiment did not detect any significant difference between *DLC2^−/−^* and *DLC2^+/+^* mice. In this regard, we could not exclude the possibility that the metabolic cage experiment might not be sensitive enough to detect the small difference in the amount of food intake which may cause the observed phenotype in body weight. Also we could not exclude the fact that *DLC2^−/−^* mice might be more active and consumed more calories than *DLC2^+/+^* mice.

It was demonstrated that DLC2 localized in the mitochondria and might play a role in lipid transport [7]. In addition, it has been suggested that the START domain containing protein, StAR, is an essential component in steroid hormone production in translocation of the cholesterol from the outer membrane to the inner membrane of the mitochondria in steroidogenic cell [27]. Since DLC2 is a START domain containing protein and is able to localize in the mitochondria, it will be of interest to study the physiological function of DLC2 in relation with the lipid metabolism as well as steroid hormone biosynthesis.

DLC2 was shown to be underexpressed in HCC [1], [2], [28] and have tumor suppressor functions including suppression of cell proliferation, Ras-induced colony formation and anchorage-independent growth [1], [2]. One of our primary aims in generating the DLC2-deficient mice was to investigate the tumor suppressor role of DLC2 in vivo. We found that DLC2 deficiency did not predispose to the formation of HCC nor aggravate DEN-induced hepatocarcinogenesis. Again, the tumor suppressor function of DLC2 function may be compensated by DLC1. Since DLC1-deficient mice dies at embryonic stage, it would be interesting to see if hepatocyte-specific deficiency of DLC1 may predispose such mice to the development of HCC and to delineate the role of DLC2 deficiency in the development of HCC in these mice. It was demonstrated that loss of p53 would accelerate hepatocarcinogenesis in transgenic mice overexpressing c-myc [29]. It would be informative to cross DLC2-deficient mice with other knockout mice of tumor suppressor genes, such as p53 and/or transgenic mice overexpressing oncogenes such as c-myc and TGF-alpha [30]. Alternatively, the newly developed system by Zender et al. could be exploited to study the tumor suppressor role of DLC2 in hepatocarcinogenesis [31]. In that system, p53-deficient liver progenitor cells could be infected with a combination of ecotropic retroviruses carrying c-myc, RAS, AKT and DLC2. This will enable us to further investigate whether DLC2 has tumor suppressor function in vivo.

DLC2 belongs to a member of DLC family proteins. It encodes a RhoGAP domain with high sequence homology with DLC family members, DLC1 and DLC3. In our previous studies, we demonstrated that DLC2 or DLC1 was able to inhibit the RhoA activity which resulted in down regulation of actin stress fiber formation. Furthermore, study from Kawai et al indicated that DLC3 could also inhibit the RhoA activity by its RhoGAP domain. Ectopic expression of DLC3 in HeLa cells decreased the numbers of actin stress fibers [32]. The results suggest that the functional roles of DLC1, DLC2 and DLC3 in cell lines and their downstream effectors are quite similar. In the mouse model, DLC1 depletion was embryonic lethal while our DLC2 knockout mice could survive to adulthood. This suggests that the functions of DLC2 in embryonic development may be compensated by DLC1 or DLC3. However, from our semi-quantitative RT-PCR result, there was no significant increase of either DLC1 or DLC3 in the livers, hearts and skeletal muscles of the DLC2 knockout mice. The result implies that the physiological level of DLC1 or DLC3 expression may be sufficient for the compensation of DLC2 functions. It will be interesting to generate DLC3 knockout mice to study its significance in embryonic development and examine whether its function can be compensated by other DLC members. In addition, the knockout mice data indicate that the DLC members might play different physiological roles in development. Apart from the common downstream effector (RhoA), there might be other different molecular targets being specifically regulated by a particular DLC member.

Apart from studying the tumor suppressor role of DLC2 in HCC, the DLC2-deficient mice may be useful for studying other cancer types, as downregulation of DLC2 was also observed in lung, ovarian, renal, breast, uterine, gastric, colon and rectal cancers [33].

## Supporting Information

Figure S1 Expression level of DLC1 and DLC3 in DLC2−/− and DLC2+/+ mice. Semi-quantitative PCR performed on the livers, skeletal muscles and hearts of the DLC2−/− and DLC2+/+ mice of 3 months of age showed no significant difference in the mRNA expression levels of DLC1 and DLC3. β-actin gene was used for normalization.
(0.39 MB TIF)
