# Supplementary figures and images for: Deleted in Liver Cancer 2 (DLC2) Was Dispensable for Development and Its Deficiency Did Not Aggravate Hepatocarcinogenesis
Source: PLoS One. 2009 Aug 10;4(8):e6566. doi: 10.1371/journal.pone.0006566 (PMC2718616; doi:10.1371/journal.pone.0006566)

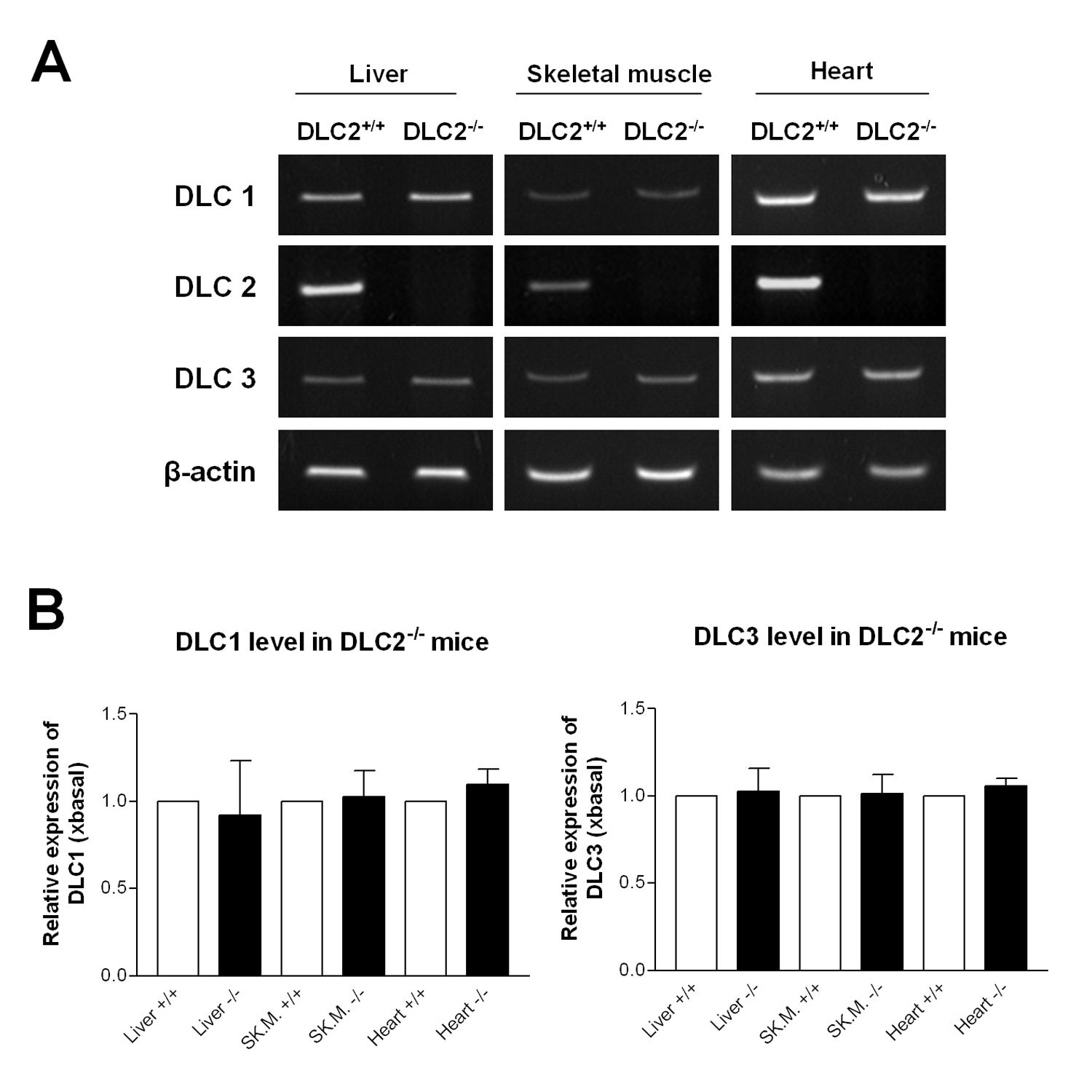

Supplement: Figure S1 — Expression level of DLC1 and DLC3 in DLC2−/− and DLC2+/+ mice. Semi-quantitative PCR performed on the livers, skeletal muscles and hearts of the DLC2−/− and DLC2+/+ mice of 3 months of age showed no significant difference in the mRNA expression levels of DLC1 and DLC3. β-actin gene was used for normalization. (0.39 MB TIF) [file pone.0006566.s001.tif]
